# Utilising a livestock model for wildlife health planning

**DOI:** 10.3389/fvets.2024.1466740

**Published:** 2024-11-27

**Authors:** Stuart Patterson

**Affiliations:** Wildlife Health, Pathobiology and Population Sciences, Royal Veterinary College, Hatfield, Hertfordshire, United Kingdom

**Keywords:** evidence-based practice, wildlife health planning, wildlife management, applied epidemiology, health outcomes, multidisciplinary teams, wildlife health and welfare, knowledge gaps

## Abstract

Health planning provides a structure for the application of epidemiological data to managed populations with the intention of maximising health and identifying targets for intervention. Whilst this is established practice in livestock health, such schemes are rarely applied to free-living wild animal populations. The health of wildlife is important for a variety of reasons including conservation, human health, and ecosystem health, and so it is recommended that a formalised health planning approach be adopted for wildlife, based upon advantages of livestock health schemes identified here. Six key strengths of livestock herd health plans are identified in that these plans are: (1) Outcome driven, (2) Structured and repeatable, (3) They can incorporate both health and welfare considerations and in doing so, establish multidisciplinary management teams, (4) Evidence-based allowing for the prioritisation of key risk factors, (5) Encompassing of both population and individual metrics, and (6) Offer the opportunity for accreditation schemes. The benefits highlighted have implications for both wildlife management and research agendas where the structured format of the health plans will highlight knowledge gaps. Challenges are acknowledged, and it is recognised that livestock health planning cannot simply be copied across to a wildlife context. However, the strengths identified are great enough that it is recommended that wildlife population health planning is developed for active management of individual populations, learning lessons from existing plans.

## 1 Introduction

Epidemiological studies play a key role in the development of animal disease control measures (1) and have therefore been crucial in the development of herd health plans (HHPs) for livestock (2). With increasing access to electronic patient records, epidemiologists have recently been able to make evidence-based recommendations with respect to the management of companion animals through projects such as Vet Compass (3). Whilst there are numerous epidemiological studies into wild animal populations, methodologies are rare for converting research into applied surveillance schemes as management tools. True surveillance differs from simply monitoring, in that the former has a requirement for health data to contribute towards plans for risk mitigation (4). It is therefore clear that more could be done to capitalise on current wildlife research in order to aid health management.

Whilst wildlife health is a popular area for research, studied for a variety of reasons, for this work to have practical application there is a need for research to lead to action (5, 6). Wildlife health may be studied for the benefit of the wild animals themselves (conservation), but other reasons for focussing on this include the potential knock-on impacts to human health (zoonotic transmission), livestock (food security), or the environment. From a One Health perspective, wildlife health has important implications for pandemic prevention (7). Poor wildlife health therefore has potential implications for conservation, ecosystem function, food security or public health (8), and so where this occurs it is likely that interventions would be required. Interventions would be expected to have an evidence base, ideally showing not just an impact on a designated risk factor, but a pathway to influencing the overall health of a population. Active health planning for a population therefore needs to be able to identify and measure factors of proven relevance to overall population health.

Whilst there is some debate around the definition of health in the human field (e.g., (9, 10)) it has long been established that this is more than merely the absence of disease. This definition is not consistently paralleled in wildlife studies with the meaning of health often assumed (11), or with a focus on a single pathogen. Ryser-Degiorgis (12) argues that whilst there may well be relevance in focussing on a single health component, population health is often multifactorial. When talking about a population’s health it is therefore important to have a clear understanding of what a good health outcome would look like. If the research interest is driven by risk of a zoonotic pathogen transmitting to humans, then it is likely to be appropriate for studies to focus on a single pathogen, whereas a multifactorial approach may well be favoured if the viability of the study population itself is of concern.

The current picture of wildlife health research only partially satisfies the requirements for the sort of applied health surveillance which can lead to meaningful interventions. This observation agrees with Stephen (13) in calling for a renewed approach to wildlife health, with a focus on providing decision-makers with tools for action. This direction of travel appears typical of a general trend in epidemiological research with Frérot et al. (14) describing an increased focus within the literature on “health,” as opposed to single pathogen and “control,” implying responsive actions.

Adapting existing methods from livestock health planning may offer a framework for developing wildlife health planning based on established protocols. This paper sets out the case for taking this approach, based around six key reasons, and makes recommendations as to how to develop wildlife population health planning. For each reason given, the role of that factor in livestock health planning is described, along with a discussion of the applicability to wildlife health.

## 2 Rationale for translating a herd-health planning approach to wildlife

### 2.1 Outcome driven

No health planning approach will succeed without a clearly defined outcome and whilst many surveillance schemes may be managed by external participants, a HHP is typically constructed around a farmer’s goals for their enterprise (15). Dairy HHPs are built around maximising milk quality and production, whilst beef and sheep equivalents will predominantly focus on meat, with economic improvements being seen as central to such approaches (16). These defined goals are essential for structuring the plan. Booker et al. (17) highlight the need for such goals as a starting point for health plans if a structured epidemiological approach is to be taken.

Analysis of the application of dairy HHPs has shown a clear positive relationship between the presence of tailored goals within a plan, and the active participation of stakeholders (18). Kristensen and Jakobsen (19), when discussing what they refer to as farmers perceived to be “irrational,” highlight the necessity for the involvement of farm owners (the stakeholders) in the setting of farm goals, and livestock health planning has increasingly moved towards this tailored approach.

In a wildlife context, the selection of defined outcomes will rarely be as straightforward as it is for a dairy herd and will need to be specific to local issues. Factors that may be considered important could include the ability of a population to maintain and transmit zoonotic infections or pathogens of livestock importance, the population’s ecosystem services, or something as fundamental as the continued existence of a population. Setting meaningful goals for wildlife health is almost certain to require the input of local stakeholders who have a good knowledge both of the populations themselves, but also of the local challenges (20–22). In practice therefore, it is important that there be careful consideration of the most appropriate methods for engaging local communities both to capitalise on their existing understanding, and to better understand their future needs (23).

The importance of incorporating clearly agreed outcomes into livestock health planning has been recognised, and there is clear merit in using this as a foundation for a wildlife model as without them both structure and participation are likely to be disadvantaged.

### 2.2 Structured and repeatable

Having established defined outcomes, the remainder of a livestock HHP will consider how best to achieve them, usually through a multi-level approach. The desired outcomes will be monitored, as well as risk factors (see below) that influence these outcomes. The approach incorporates what Cook (24) refers to as “top-level” indicators and a “drill-down” techniques. Within this system a series of key performance areas are identified which relate to the overall outcome. For example, a dairy HHP focussed on farm milk output may have top-level reporting for animal nutrition, infectious disease, cow mobility (all impacting animals’ ability to access and convert energy), reproductive health, youngstock rearing (both considering the next generation of milk producers), and milk quality itself. This list is of course not exhaustive. Each of these top-level categories is impacted by a huge range of different factors, but if the herd is performing well in one of these areas then it is an inefficient use of time to be investigating the risk factors in detail. However, when a category is performing sub-optimally, managers can “drill down” to those risk factors. Interventions are then targeted at risk-factors that appear to be impacting those selected population outcomes.

Resources for investigating wildlife health are notoriously stretched (12) and a structure such as this which only prioritises investigation and intervention into areas where there is a functional deficit would seem to be an efficient use of time and budget. Clearly identifying the most appropriate top-level categories would require careful thought, and there would be a need for research in order to create an evidence base for such plans. The clear advantages of such an approach for wildlife would include the transferability and repeatability of the approach allowing it to be locally adapted and implemented by a range of different individuals.

### 2.3 Incorporation of both health and welfare criteria within a multidisciplinary team

It is common to encounter the phrase “health and welfare” with no clear boundary established between those two different measures. This is true within a wildlife context as much as anywhere else and the two terms have their own definitions and specialists. The result of this can be siloed teams working independently of each other; inefficient in terms of both resources and the sharing of ideas. Livestock HHPs recognise that both health and welfare parameters contribute towards farm production outcomes and so incorporate both areas within typical plans. In livestock systems, a tendency has been noted for welfare investigations to focus too heavily on those welfare concerns relating to production diseases (25), and whilst this is unlikely to be a concern in a non-production context, being alert for study biases is a useful lesson.

There is therefore an opportunity to incorporate both wildlife health and wildlife welfare into wildlife health planning, thus improving both efficiency and a cohesion between practitioners. The process of developing models for dairy herd health planning has coincided with the evolution of multidisciplinary teams for managing on-farm health (26). Cook (24) talks about a change in style within veterinary practice over recent decades, moving from the “physician” model through to the “facilitator” model, whereby veterinary involvement in health has become about bringing together multidisciplinary teams rather than simply treating individual animals. Both Cook (24) and Kelly et al. (26) partially attribute these shifts to an increasing tendency to focussing on production (outcome) and population level management with larger herds.

Given the changes that have been seen in livestock health planning, it can be expected that the development of multidisciplinary teams for managing the health of wildlife populations would better facilitate interactions between disciplines and incorporate metrics of both health and welfare in their own rights. Currently the number of projects incorporating both health and welfare in wildlife populations is low.

### 2.4 Evidence-based approach

The top-level system that has been described here is built upon a body of livestock health research. In cases where the top level figures for clinical mastitis, for example, in a herd are considered unacceptably high, then identification of the pathogens involved may be a possible next step. Bacteria, such as *Escherchia coli*, are considered to be of environmental origin as opposed to being transmitted cow to cow, and so interventions targeting contamination of the environment would be favoured over addressing cow to cow transmission (27). Or in cases of poor mobility, investigations into the anatomical location of pathologies will lead investigators towards likely causes and therefore the interventions with the greatest chance of success (28). In both examples there is a chain of established evidence linking interventions to risk factors to outcomes.

The key advantages of this approach are that it does not require continual analysis of all potential risk factors (which would be labour intensive, expensive, and probably unrealistic), and it ensures that any data that is being collected and analysed is related to a defined health objective and is therefore meaningful. Whilst these are advantageous in livestock health work, these advantages are magnified in wildlife where observation and sampling is likely to be far more difficult. Without a structured approach to health planning in wildlife currently there are a whole range of analyses carried out, but the association between each analysis and an overall goal is rarely evidenced. By utilising this approach, risk factor analysis could be implemented to ensure that factors forming part of a surveillance scheme were those that were truly associated with desired outcomes. Common approaches in livestock health consider not just the presence or absence of a component, but also implement intervention thresholds [e.g., (29)], and this may be something for the future once the initial stages of wildlife health planning are established. As well as merely identifying those key measurements required, this then means that the value of interventions can be better understood and investments justified.

This does present a problem for translating livestock HHP approaches to wildlife as in the majority of cases the causal relationships between desired outcomes and risk factors have not been proven. Whilst this clearly offers a challenge, it does also mean that the proposed structure of a wildlife health plan would effectively become scaffolding for research priorities. This could be of use to both funding bodies as they will be able to see immediately a pathway to impact for proposed research, and to early career researchers looking to establish meaningful projects.

### 2.5 Encompassing both population and individual metrics

Population health statistics come in two forms; those that consider the population as the unit of interest, or those that collate data on individuals. The difference between these two approaches is often not highlighted, but both have advantages and disadvantages. Population-based measures may be much more useful when the desired health outcome from a population is, for example, an environmental impact. In such cases, what is important is not the proportion of individuals carrying out the desired activity, but the overall impact of the population. With increasing sizes in dairy herds, some movement has been seen towards health measures that reflect a population outcome without information about the individuals, for example the use of bulk tank milk samples (aggregate data) for disease testing (30). In such cases the reported statistic gives useful information about the final output, but the result could be swayed by one extremely heavily infected individual, and it would not be possible to distinguish this from low-level infections throughout the herd.

Livestock HHPs routinely use both population and individual measures and therefore can be used as a template for how to handle both. Understanding the origin of the data and being familiar with what interpretations can and cannot be made on the basis of a measurement are clearly key to being comfortable utilising both types of data. Health measures based on population parameters are utilised in both livestock and human health but are rare in wildlife health studies where population health tends to be described as a collection of individual health statistics. Aggregate data, for some observations, may be easier to obtain in a wildlife setting than individual samples, and so a recognition of how to incorporate such data in wildlife health planning would be a valuable lesson learned from livestock HHPs.

### 2.6 Accredited schemes

The final consideration mentioned here is that of the use of livestock health plans as essential components of accreditation schemes. This may not be the way in which every wildlife health practitioner wants to go, but there is the possibility of being able to use a structured wildlife health plan as evidence for the success of wildlife projects. In the United Kingdom, farm assurance schemes have increased the uptake of HHPs with benefits including increased milk quality and control of antimicrobial residues (31). There is therefore evidence in livestock that health planning has played a role in improving the health of farmed animals, and that participation has often been driven by the requirements of assurance schemes (32).

The formal structure of livestock HHPs, were it to be adopted for wildlife, could therefore have the dual benefits of incentivising participation from wildlife managers, and providing confidence for funding agencies. Financing wildlife health interventions can be very challenging, and being able to evidence to investors that their money will be used in an effective manner has great value (33). Environmental accreditation schemes have the potential to become very complex and participation in them should not be allowed to be the central determinant of the desired health outcomes of a wildlife health plan. However, if managed carefully formal wildlife health plans could be integrated into accreditation schemes with benefits in terms of participation, funding and recognition.

## 3 Discussion

The factors laid out above highlight the advantages of adopting a structured approach to wildlife health planning, building upon existing techniques for livestock health planning. Basing the wildlife model on existing livestock health structures offers the opportunity to reflect on current practices, learning lessons from what works well. Key advantages highlighted include structure, a framework for linking metrics (and interventions) to population outcomes, and a system which would highlight research needs.

Whilst it would be naïve to suggest that planning tools from livestock health could simply be copied directly across to a wildlife population, the broad principles described here would appear valuable enough to justify the effort involved in adapting processes. The current model of livestock HHPs was not struck upon immediately and is one which has evolved over time. A logical next step would therefore be to bring together a range of livestock practitioners and wildlife health professionals in order to highlight the most appropriate building blocks for a wildlife planning model, and to identify knowledge gaps that are important to address.

The requirement for clearly agreed wildlife health outcomes from the outset of this process stands out as key from these discussions as this feeds into so many of the other factors discussed. It is difficult to envision a scenario where health targets can be established for a population without integration of stakeholder inputs. Seeing wildlife population health as a context-specific set of goals rather than a rigid state will help to make health management plans more adaptable. Whilst a need will remain for single-issue health investigations, a model for tailored wildlife health planning will be essential for a move towards a more holistic view of health for wildlife species.

Livestock health planning and evidence based veterinary medicine have offered platforms for both veterinary practitioners and epidemiologists to contribute to wider teams managing livestock health. It is therefore strongly recommended that these principles now be extended to wildlife health.

## Data Availability

The original contributions presented in the study are included in the article/supplementary material, further inquiries can be directed to the corresponding author.
